# Neutrophils protect lymphoma cells against cytotoxic and targeted therapies through CD11b/ICAM-1 binding

**DOI:** 10.18632/oncotarget.20350

**Published:** 2017-08-18

**Authors:** Taghreed Hirz, Eva-Laure Matera, Kamel Chettab, Lars Petter Jordheim, Doriane Mathé, Anne Evesque, Justine Esmenjaud, Gilles Salles, Charles Dumontet

**Affiliations:** ^1^ Anticancer Antibody Team, INSERM U1052, CNRS UMR 5286, Cancer Research Center of Lyon, University of Lyon, Lyon, France; ^2^ Hospices Civils de Lyon, Department of Hematology, Pierre-Benite, France; ^3^ Université Claude Bernard Lyon-1, Lyon, France; ^4^ ProfileXpert, Lyon, France; ^5^ Laboratory of Hematology, Hospices Civils de Lyon, Pierre-Bénite, France

**Keywords:** neutrophils, lymphoma, chemotherapy, targeted therapy, cell-cell interactions

## Abstract

Innate immune cells constitute a substantial proportion of the cells within the tumor microenvironment. Besides the contribution of the microenvironment to tumor proliferation and survival, there is direct evidence that interactions between tumor cells and their microenvironment alter sensitivity to anti-cancer agents. Neutrophils, a key player in the innate immune system, have been less studied than many other immune cells regarding their impact on cancer cell response to anti-cancer agents. In our 2D and 3D coculture systems, human neutrophils and differentiated HL60 cells attenuated the sensitivity of various lymphoma cell lines to several anti-cancer agents, including targeted therapies. Neutrophil-induced protection was dependent on cell-cell interaction between CD11b and ICAM-1 expressed by neutrophils and B cells, respectively and was shown to be Mcl-1-dependent. The protective effect of neutrophils was validated *in vivo* using immune-compromised mice inoculated with human NHL with our without neutrophils then followed by treatment with chemotherapy. Similar findings were made on primary cells purified from patients with chronic lymphocytic leukemia, treated with fludarabine or targeted agents in the presence of autologous neutrophils. In a clinical study, patients with non-Hodgkin's lymphoma with increased neutrophil counts displayed a reduced response rate to therapy. These findings reveal a novel protective mechanism of neoplastic B cells involving innate immune cells which could be pharmacologically targeted to enhance the antitumor effect of therapy.

## INTRODUCTION

Non-Hodgkin's lymphoma (NHL) is the most common cancer of the lymphatic system, with a large number of disease subtypes. Several studies have described interactions between lymphoma cells and non-malignant cells within the bone marrow or lymph nodes, including follicular dendritic cells [1, 2], human bone marrow stromal cells [3] and derived nurse-like cells [4]. There is a clear impact of the adaptive immune compartment on the outcome of patients with lymphoma [5], but much less data exist regarding the role of the innate immune cells. The presence of polarized M2 macrophages has been shown to be an independent predictor of poor prognosis in the context of NHL [5] as M2 macrophages are known to secrete immunosuppressive cytokines inducing tumor cell growth [6]. Few data are available regarding the interactions between cancer cells and neutrophils which are key players in the innate immune system.

Although neutrophils are traditionally considered in the context of their antibacterial functions, it is becoming increasingly clear that tumor-associated neutrophils (TANs) and their myeloid precursors (granulocytic myeloid-derived suppressor cells (G-MDSC)) in the spleen, bone marrow and blood play an important role in cancer biology [7–12]. They have been proposed as key mediators of malignant transformation, tumor progression, angiogenesis and in the modulation of antitumor immunity [13–18]. Tumor-associated neutrophils have been reported to facilitate resistance of hepatocellular carcinoma to sorafenib via the recruitment of macrophages and T-regulatory cells [19]. Manfroi et al. recently reported that IL8 produced by diffuse large B cell NHLs (DLBCL) could recruit APRIL-producing blood neutrophils and that high APRIL expression in DLBCL correlates with reduced patient survival [20]. Spontaneous neutrophilic infiltrates are highly variable depending on NHL subtypes, with strong infiltrates reported in cutaneous T-cell lymphomas [21]. However neutrophils may be recruited through their Fc-gamma receptors upon administration of therapeutic antibodies such as rituximab [22].

Regarding the effect of different immune cells in regulating tumor response to cancer therapies, the potency of macrophages and inflammatory monocytes to induce tumor progression and the mechanisms by which they regulate treatment response are well defined [23–26]. However, the potential role of neutrophils in regulating tumor response to anticancer treatment is not known.

In this study, we investigated the mechanisms by which circulating neutrophils regulate the response of NHL and fresh primary leukemic cells to various types of cytotoxic compounds as well as targeted agents.

## RESULTS

### Freshly purified human neutrophils protect lymphoma B-cells against the cytotoxicity of chemotherapeutic agents through direct contact

**Figure 1:**
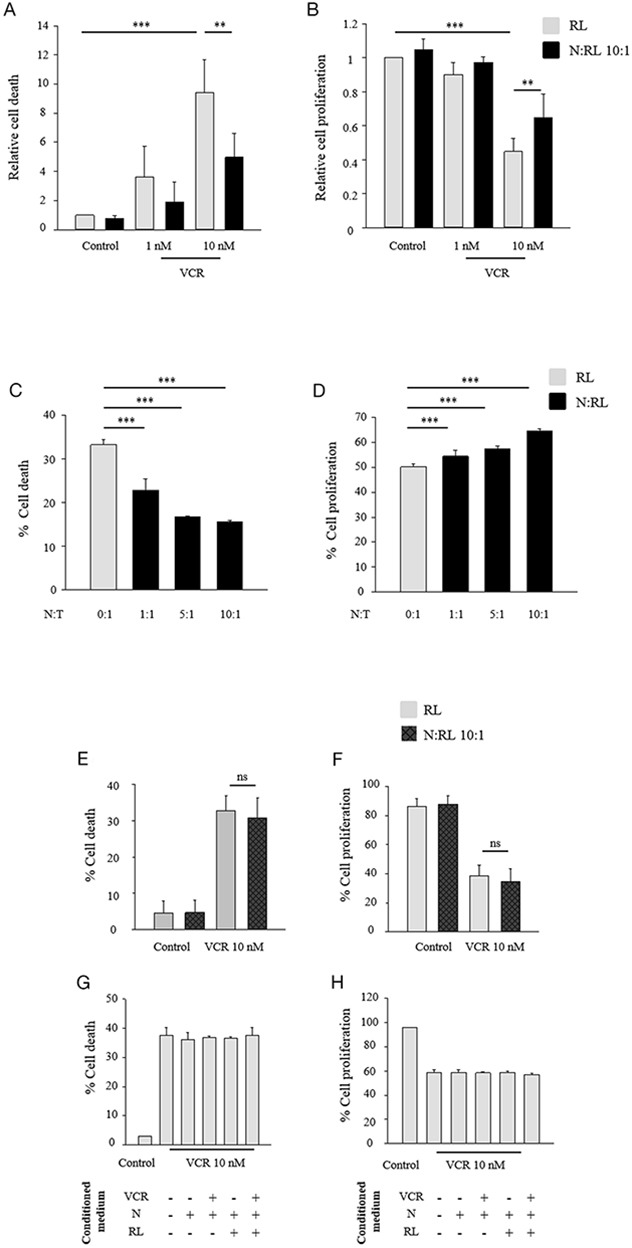
Neutrophils protect lymphoma B-cells against vincristine in a ratio-dependent manner induced by cell-cell contact **(A-D)** CFSE-labeled lymphoma B-cells were cultured alone or together with neutrophils (N) at different N:T ratios, in the presence or absence of 10 nM vincristine (VCR). After 48 h of incubation, cells were labeled with anti human-CD19 then resuspended in DAPI (2 μg/ml) followed by flow cytometric analysis. Cell death (A, C) and cell proliferation (B, D) of CD19 positive population were measured using DAPI and CFSE assays, respectively. Data are expressed as mean ± SD of at least three independent experiments performed in triplicates. The data are presented relative to the control. Ratio-dependent figures (C-D) represent data of one experiment performed in triplicate as mean ± SD. **(E-F)** CFSE-labeled RL cells and neutrophils were co-cultured on opposite sides of porous PET inserts. Neutrophils (N) were placed inside the inserts whereas CFSE-labeled RL cells were placed in the wells, or to inserts with complete RPMI medium as a control. VCR at 10 nM was incorporated at the initiation of culture on CFSE-labeled RL cells. After 48 h of incubation, CFSE-labeled RL cells were resuspended in DAPI (2 μg/ml) followed by flow cytometric analysis. Cell death (E) and cell proliferation (F) were measured using DAPI and CFSE assays, respectively. Data are expressed as mean ± SD of three independent experiments. (G-H) CFSE-labeled RL cells were cultured in the presence of 10 nM VCR in a complete RPMI medium, or with conditioned mediumfor 48 h.. then CFSE-labeled RL cells were resuspended in DAPI (2 μg/ml) followed by flow cytometric analysis. Cell death (G) and cell proliferation (H) were measured using DAPI and CFSE assays, respectively. Data are expressed as mean ± SD of one experiment performed in triplicate. One-way ANOVA statistical test was used for multiple comparisons applying Holm-Sidak method. **p≤ 0.01, ***p≤ 0.001, ns= not significant.

To investigate the effect of neutrophils on lymphoma cells, purified total neutrophils from peripheral blood of healthy donors were co-cultured with different human NHL cell lines (RL, Granta, Raji and Es-moult) in the presence or absence of various anti-cancer agents (vincristine (VCR), doxorubicin, bortezomib, cisplatin, and mafosfamide) at different concentrations. Results in Figure 1A and 1B show that vincristine induced cell death (Figure 1A) and inhibited cell proliferation (Figure 1B) in a dose-dependent manner in RL cells. Vincristine-dependent effect on both parameters was significantly reduced in the presence of neutrophils. Comparable results were obtained using other lymphoma B cell lines such as Granta, Es-moult and Raji exposed to vincristine at different concentrations (Supplementary Figure 1).

Similar results supporting the protective effect of neutrophils were obtained with RL cells exposed to doxorubicin (10 nM, 1 μM), bortezomib (10 nM, 15 nM), cisplatin (5 μM, 25 μM), or mafosfamide (50 μM) (Supplementary Figure 2). Cisplatin and mafosfamide did not induce RL cell death at these concentrations but caused a significant inhibition of cell proliferation. We did not observe any impact of the different chemotherapeutic agents on neutrophils viability at the concentrations used. Overall, these results show that neutrophils exert a protective effect on several NHL models against a variety of conventional cytotoxic compounds *in vitro*.

We further determined whether the protective effect of neutrophils was influenced by the neutrophil:tumor (N:T) cell ratio. CFSE-labeled RL cells were co-cultured with different ratios of neutrophils in the presence of vincristine at 10 nM. Figure 1C and 1D show that the protective effect was correlated with the N:T cell ratio and could be observed at a ratio as low as 1:1.

We next examined whether the protective role of neutrophils was due to the sequestration of chemotherapy. RL cells were incubated alone or with neutrophils in the presence or absence of doxorubicin. The amount of doxorubicin accumulating in RL cells was examined by flow cytometry. Results show that the uptake of doxorubicin by RL cells was not reduced in the presence of neutrophils although neutrophils also showed an uptake of doxorubicin (Supplementary Figure 3).

The requirement of cell-cell interaction for the protective effect of neutrophils on tumor cells was explored using transwell experiments and neutrophil-conditioned media. The presence of neutrophils inside the inserts did not modify the effect of vincristine on RL cell death (Figure 1E) or cell proliferation (Figure 1F). Similar results were obtained in experiments in which RL cells were cultured for 48 hours with conditioned medium (CM) harvested after 2 hours from cultures of neutrophils alone or exposed to RL cells and in the presence or absence of vincristine (Figure 1G and 1H). Similar results were obtained using CM harvested at different periods of time (data not shown). These results indicate that the neutrophil-induced protection of lymphoma B-cells against vincristine requires a direct contact between the two cell types.

### Blocking cell interaction suppresses the protective effect of neutrophils which is dependent on Mcl-1

Since cell-cell contact was determined to be important for neutrophil-mediated protection of RL cells, we examined the expression of different surface markers on neutrophils cultured alone or with RL in the presence or absence of vincristine. Results in Supplementary Table 1 show that neutrophils spontaneously express CD11a, CD11b, CD18, CD32 and CD66b at high levels with lower expression of CD64. Exposure to vincristine did not modify the expression level of these activation markers whereas the co-culture of neutrophils with RL increased the expression of CD11b, CD18, CD64 and CD66b (6.4-fold, 3.8-fold, 3-fold and 4.5-fold increases, respectively) and decreased CD32 (2-fold decrease), whereas the expression of CD11a remained unchanged.

**Figure 2:**
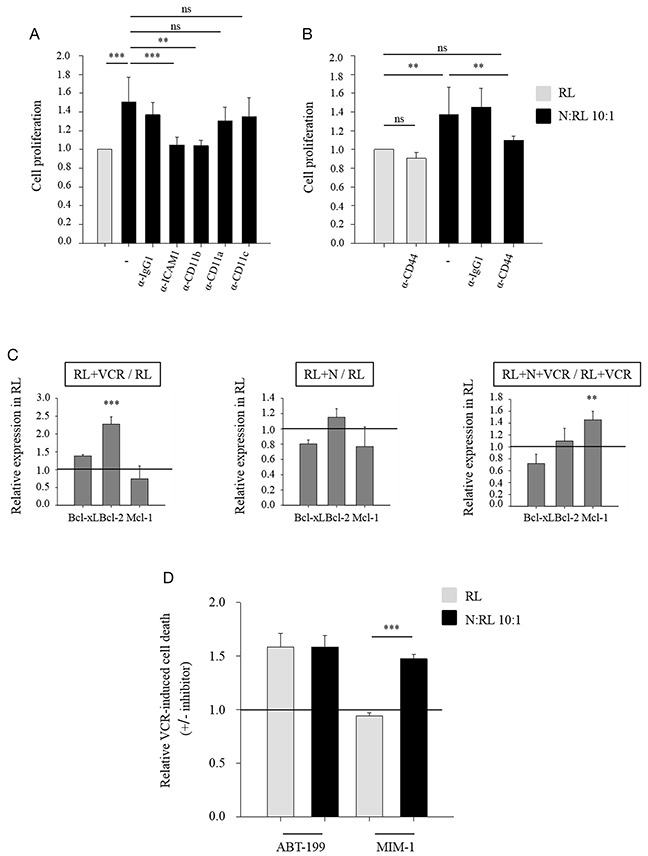
ICAM1, CD11b and CD44 are implicated in neutrophil-induced protection depicted as Mcl-1-dependent **(A)** CFSE-labeled RL cells were blocked against ICAM1 (12 μg/ml), CD11a or CD11c (10 μg/ml) and neutrophils were blocked against CD11a, CD11b or CD11c (10 μg/ml) for 1 hour. Anti-IgG1 served as a control. CFSE-labeled RL cells were cultured alone or together with neutrophils (N) at N:RL ratio 10:1 in the presence of 10 nM VCR for 48 h. Cell proliferation of CD19 positive population was measured using CFSE assay followed by flow cytometric analysis. Data are expressed as mean ± SD of at least four independent experiments performed in triplicates. The data are presented relative to the control. **(B)** CFSE-labeled RL cells were cultured alone or together with neutrophils in the presence or absence of CD44 blocking antibody (1 μg/ml) treated with 10 nM VCR. Cell proliferation of CD19 positive population was measured using CFSE assay followed by flow cytometric analysis. Data are expressed as mean ± SD of three independent experiments performed in triplicates. The data are presented relative to the control. **(C)** Relative expression of Bcl-2 family members (Bcl-xL, Bcl-2, Mcl-1) in RL cells. RL cells were cultured alone or together with neutrophils at N:T ratio 10:1, in the presence or absence of 10 nM VCR. After 48 hours of incubation, the cells were collected and labeled with anti-human CD19 thenfixed and permeabilized. Later, the intracellular labeling of anti-apoptotic Bcl-2 family members was performed by flow cytometry. Data are expressed as mean ± SD of at least three independent experiments performed in triplicates. **(D)** RL cells were cultured alone or together with neutrophils at N:T ratio 10:1, in the presence or absence of 10 nM VCR. ABT-199 (10 nM) or MIM-1(10 μM) inhibitors were added to the culture system. After 48 h of incubation, cells were labeled with anti human-CD19 then resuspended in DAPI (2 μg/ml) followed by flow cytometric analysis. Cell death of CD19 positive population was measured using DAPI assay. Data are expressed as mean ± SD of three independent experiments performed in triplicates. One-way ANOVA statistical test was used for multiple comparisons applying the Holm-Sidak method. **p≤ 0.01, ***p≤ 0.001, ns= not significant.

It has previously been shown that CD11a, CD11b and CD11c integrins at the surface of neutrophils interact with the adhesion molecule ICAM-1 [27–29] which is involved in the regulation of cell proliferation [30, 31]. To determine whether these receptors were implicated in the protective effect of neutrophils, we used blocking antibodies against these surface antigens and ascertained their effect on RL cell growth and cell death in the presence of vincristine. Blocking antibodies recognizing ICAM-1 expressed by RL or CD11b expressed by neutrophils inhibited neutrophil-induced protection on RL cell proliferation against vincristine, whereas blocking antibodies recognizing CD11a or CD11c (expressed both by RL cells and neutrophils) had no effect (Figure 2A). No effect was obtained on RL cell death (data not shown). These results underline the importance of CD11b/ICAM-1 binding in neutrophil-mediated protection. No effect on RL cell growth was obtained by blocking direct contact between both cell types in the absence of VCR (Supplementary Figure 4A and 4B).

In order to obtain insight into the changes induced in RL cells by neutrophils and vincristine, we used a transcriptomic approach on RL cells. Among the different genes found to be up-regulated in RL cells obtained from co-cultures with neutrophils and vincristine (Supplementary Table 2), CD44 was among the most strongly over-expressed. As CD44 is another adhesion molecule involved in regulating cell proliferation [32–34], we used blocking antibody recognizing CD44 and observed abrogation of the neutrophil-induced protection of RL cell proliferation in the presence of vincristine (Figure 2B).

Results of the transcriptomic analysis of RL cells showed a regulation in multiple genes involved in several pathways such as Jak/Stat pathway, apoptosis and cell death (Supplementary Figure 5). Thus we examined the effect of neutrophils on the expression by NHL cells of proteins known to regulate apoptosis of tumor cells, such as anti-apoptotic Bcl-2 family members. For these experiments, RL cells were cultured alone or with neutrophils in the presence or absence of 10 nM vincristine. Vincristine significantly increased the content of Bcl-2 in RL cells, whereas Bcl-xL and Mcl-1 expressions did not change significantly (Figure 2C: relative expression, Supplementary Figure 6: absolute expression MFI: mean fluorescence intensity). The co-incubation of neutrophils and RL cells induced a slight but non-significant down-regulation of Bcl-xL and Mcl-1 contents in RL. Conversely, the effect of neutrophils on RL cells in the presence of vincristine was a significant increase in Mcl-1 expression (Figure 2C: relative expression, Supplementary Figure 6: absolute expression MFI: mean fluorescence intensity). Thus the effect of neutrophils on the anti-apoptotic protein content of NHL cells was different when these NHL cells were simultaneously exposed to chemotherapeutic agents.

To determine the role of these anti-apoptotic proteins in the protective effect of neutrophils, co-cultures were performed in the presence or absence of small molecule inhibitors against Bcl-2 (ABT-199) and Mcl-1 (MIM-1). ABT-199 significantly enhanced the cytotoxic effect of vincristine as determined by the increase in RL cell death (Figure 2D: relative cell death, Supplementary Figure 7A: absolute value of cell death), and this effect was not influenced by the presence of neutrophils. On the other hand, MIM-1 significantly reduced the protective effect of neutrophils on vincristine-induced RL cell death (Figure 2D: relative cell death, Supplementary Figure 7B: absolute value of cell death) suggesting that the protective effect of neutrophils is Mcl-1 dependent.

### Mcl-1 upregulation via IL6/Jak/Stat pathway

Our data indicated that RL cells, by direct contact with neutrophils, induced the latter to undergo activation as determined by the increase in the expression of several activation markers (Supplementary Table 1). We examined whether activated neutrophils in our co-culture conditions release IL6 cytokine that may have a direct effect on Mcl-1 induction through the activation of the Jak/Stat pathway as previously described [35]. IL6 concentrations were quantified in the supernatant of our different culture conditions using ELISA and were detectable (0.32 pg/ml) only when RL cells were exposed to vincristine in the presence of neutrophils. Then we detected the phosphorylation level of Stat-3 in RL cells using flow cytometry at different times. For these experiments, RL cells were cultured alone or with neutrophils in the presence or absence of 10 nM vincristine. We did not observe a phosphorylation at Tyr705 when RL cells were cultured alone or exposed to vincristine (Supplementary Figure 8). Conversely, the presence of neutrophils induced the phosphorylation of Stat-3 at Tyr705 in RL lymphoma cells as early as 5 min and in a time-dependent manner to reach its maximum level at 30 min (Supplementary Figure 8).

**Figure 3:**
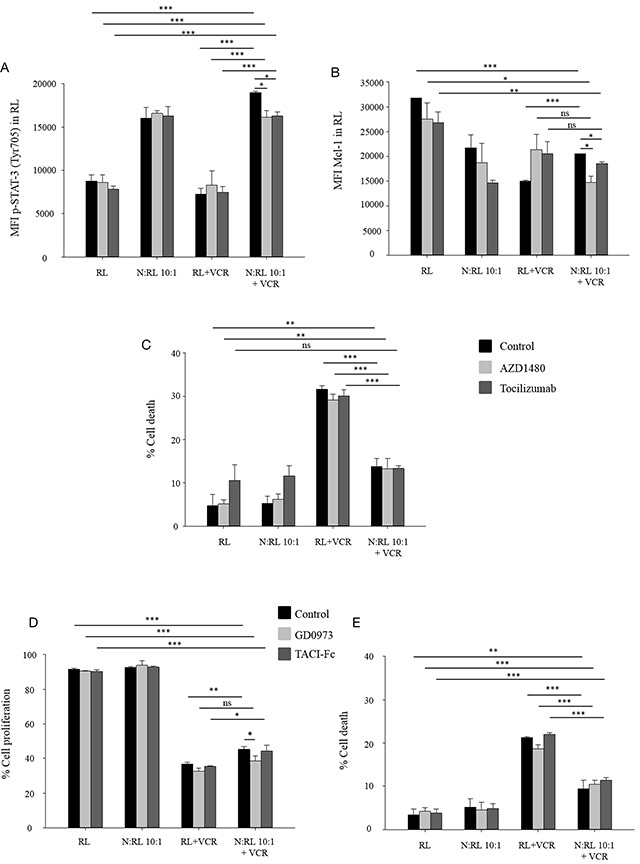
IL6/Jak/Stat pathway induces Mcl-1 upregulation **(A-C)** RL cells were cultured alone or together with neutrophils at N:T ratio 10:1 in the presence or absence of AZD1480 (1 μM) or tocilizumab (2 μg/ml) treated with 10 nM VCR. Cells were collected and labeled with anti-human CD19 then fixed and permeabilized to measure, the phosphorylation level of Stat-3 at Tyr705 at 30 minutes (A) and level of Mcl-1 expression at 48 h (B) in CD19 positive population by flow cytometry. Data are expressed as mean ± SD of three independent experiments. (C) After 48 h of incubation, cells were collected and labeled with anti-human CD19 then resuspended in DAPI (2 μg/ml) followed by flow cytometric analysis. Cell death of CD19 positive population was measured using DAPI assay. Data are expressed as mean ± SD of three independent experiments. **(D-E)** CFSE-labeled RL cells were cultured alone or together with neutrophils at N:T ratio 10:1 in the presence or absence of GD0973 (10 nM) or TACI-Fc (100 ng/ml) treated with 10 nM VCR for 48 h. Latercells were collected and labeled with anti human-CD19 then resuspended in DAPI (2 μg/ml) followed by flow cytometric analysis. Cell proliferation (D) and cell death (E) of CD19 positive population were measured using DAPI and CFSE assays, respectively. Data are expressed as mean ± SD of three independent experiments. *p≤ 0.05, Student's *t* test.

We then examined whether the IL6/Jak/Stat pathway lead to Mcl-1 upregulation and subsequently to neutrophil-induced protection of RL cell death. AZD1480 (Jak2 inhibitor) or tocilizumab (a humanized monoclonal antibody against the IL6 receptor) were added to our co-culture system to ascertain their effect on Stat-3 phosphorylation, Mcl-1 upregulation and cell death. We found that neutrophils co-cultured with RL cells induced p-Stat-3 after 30 minutes of incubation (Figure 3A). The presence of AZD1480 or tocilizumab significantly reduced p-Stat-3 in RL cells co-cultured with neutrophils in the presence of 10 nM VCR. Furthermore, blocking JAK2 activity or blocking IL6R significantly decreased the upregulation of Mcl-1 expression induced by neutrophils in the presence of 10 nM VCR (Figure 3B). Conversely, AZD1480 and tocilizumab had no effect on the protective role of neutrophils against VCR-mediated cell death (Figure 3C).

It has previously been shown by Holland *et al.* [30] that the Raf/MAPK pathway is activated downstream of the ICAM-1 receptor. In addition, Gregoire and his colleagues [36] reported that neutrophils reversed serum deprivation-induced growth arrest of B-lymphoma cell lines through direct contact mediated by BAFF/APRIL pathway. Also, the activated neutrophils are known to produce the soluble B cell-activating factor BAFF and the proliferation-inducing ligand (APRIL) [37] that have been shown to trigger lymphoma B-cell survival through their receptors such as TACI [38, 39]. To detect whether these pathways are implicated in neutrophil-mediated protection of RL cells against chemotherapy, we introduced a MEK inhibitor (GD0973) and a blockade of BAFF/APRIL activity (TACI-Fc) to our co-culture system. Neutrophils co-culture significantly induced RL cell proliferation in the presence of vincristine, an effect that was slightly reduced in the presence of GD0973 but not TACI-Fc (Figure 3D). No effect was obtained by adding these inhibitors on neutrophil-induced protection of RL cell death (Figure 3E).

### Neutrophil-like HL60*diff* cells protect RL lymphoma cells against vincristine in 3D culture

We aimed to investigate the effect of neutrophils on lymphoma cells using 3-Dimensional (3D) culture. Given the short half-life of neutrophils *in vitro*, experiments with fresh human neutrophils were not conclusive. We thus chose to use HL60 cells differentiated in the presence of DMSO and retinoic acid (HL60*diff*) which showed a strong increase in CD11b and CD38 expression indicative of granulocytic differentiation, in addition to morphological changes such as multi-lobed nuclei (Supplementary Figure 9A and 9B). HL60*diff* stopped proliferating after five days of induction of differentiation and exhibited phagocytic activity (data not shown). When RL cells were co-cultured with HL60*diff* cells in a classical 2D system at a ratio of 10 HL60*diff*: 1 RL cell, a protective effect against vincristine was observed which was similar to that induced by fresh neutrophils (Supplementary Figure 9C and 9D).

**Figure 4:**
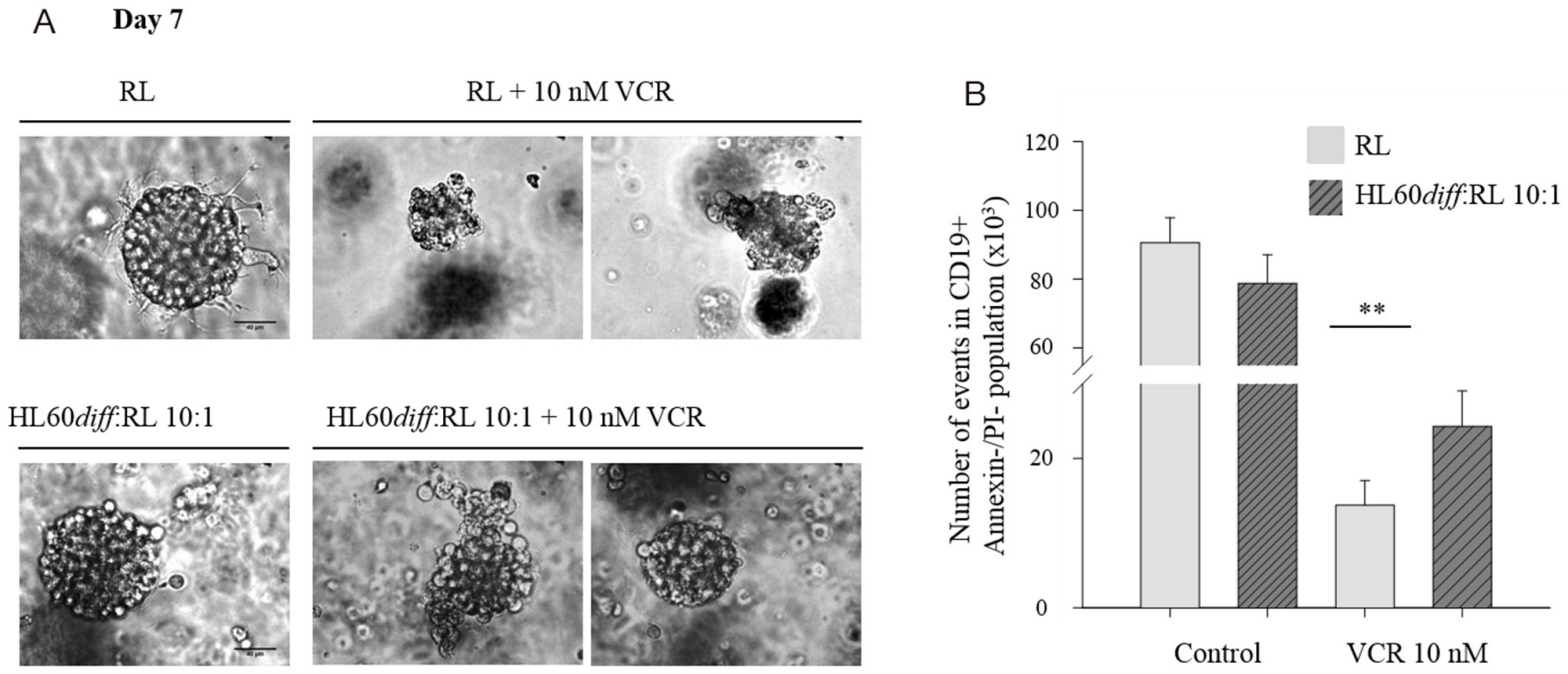
Neutrophil-like HL60*diff* cells protect RL lymphoma cells against vincristine in 3D culture RL cells were cultured alone or together with HL60*diff* cells at HL60*diff* :RL ratio 10:1 for 7 days in BD matrigel. On day 5, VCR was added at a concentration of 10 nM. **(A)** Pictures were taken on day 7 with a DMI3000 fluorescent microscope at magnification 40x, representing 15 spheroids observed microscopically in several experiments. Scale bar 40 μm. **(B)** Spheroids were dissociated on day 7 using PBS-5mM EDTA and cells were labeled with anti-human CD19 and anti-human CD38. The number of events in CD19+ Annexin V-/PI- population was measured by double staining with annexin V–FITC and PI, followed by flow cytometric analysis. Data are expressed as mean ± SD of three independent experiments performed in triplicates. One-way ANOVA statistical test was used for multiple comparisons applying Holm-Sidak method. **p≤ 0.01

RL cells were cultured alone or with HL60*diff* in Matrigel in the presence or absence of vincristine. As shown in Figure 4A, vincristine induced RL spheroid dissociation. The presence of HL60*diff* cells reduced the sensitivity of RL spheroids to vincristine as determined by the size of spheroids after seven days of culture. These results were confirmed by Annexin V/PI staining of the cells obtained after spheroid dissociation, with a significantly higher proportion of CD19 viable cells (CD19 positive, Annexin V negative/PI negative) in the presence of HL60*diff* cells (Figure 4B).

### Neutrophils attenuate the sensitivity of RL cells to vincristine *in vivo*

**Figure 5:**
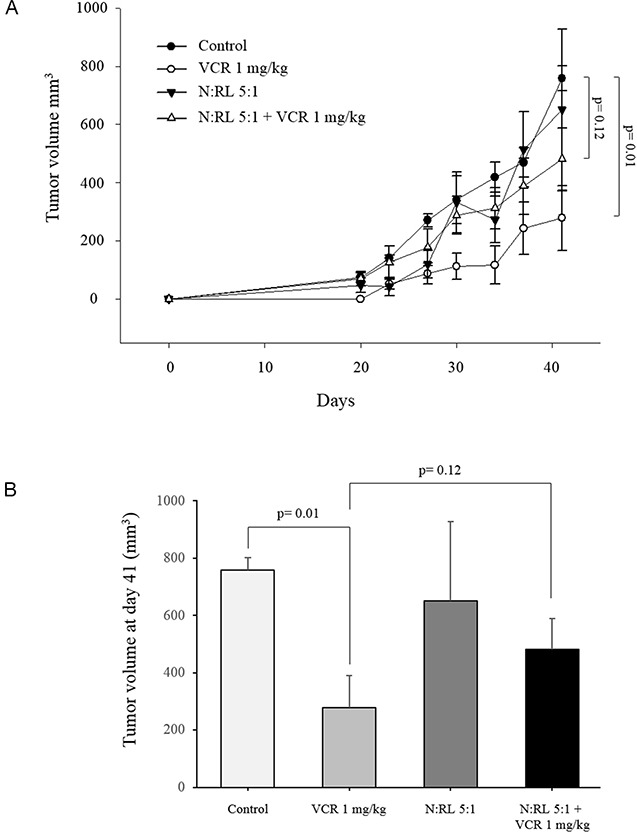
Neutrophil-induced protection on RL cells *in vivo* Mice (5 mice/group) were injected subcutaneously with 3×10^6^ RL cells alone or together with freshly purified human neutrophils (N) at N:T ratio 5:1. VCR was injected intraperitoneally to mice at a dose of 1 mg/kg at the same day. **(A)** Tumor growth was monitored by caliper measurement of the tumor volume. **(B)** Bar graphs represent the tumor volume at day 41. Student's *t* test used for group comparisons.

We examined the effect of neutrophils on the sensitivity of RL cells to vincristine *in vivo*. Purified human neutrophils from healthy donors and RL cells were co-injected subcutaneously into SCID/CB17 mice then mice were treated with vincristine on the same day. Results in Figure 5 show that vincristine significantly reduced RL tumor growth compared to control mice. In mice treated with vincristine, the inoculation of neutrophils together with RL cells (N:RL 5:1) reduced the sensitivity of RL to vincristine resulting in significantly enhanced tumor growth. Similar results were obtained when neutrophils inoculated together with RL cells at the ratio of N:RL 10:1 (data not shown). These data confirm the protective role of neutrophils previously observed *in vitro* (2D and 3D).

### Autologous neutrophils protect primary chronic lymphocytic leukemic cells against anti-leukemic agents

**Figure 6:**
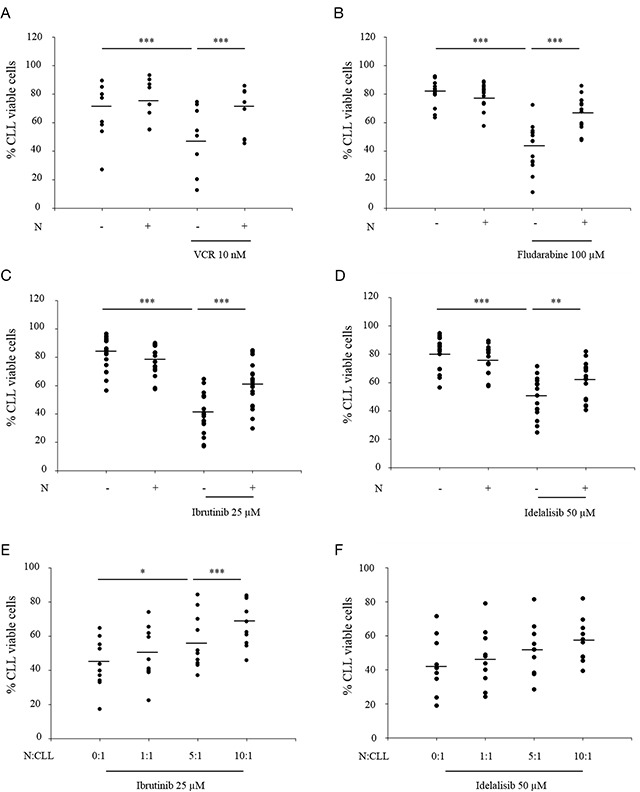
Autologous granulocytes protect primary leukemia cells against cancer therapy in a ratio-dependent manner **(A-D)** Blood was collected from patients diagnosed with chronic lymphocytic leukemia (CLL). Primary leukemic cells (CLL cells) were isolated and cultured alone or together with autologous neutrophils (N) at N:CLL ratio 10:1 for 24 h, in the presence or absence of (A) 10 nM VCR (8 patients), (B) 100 μM fludarabine (14 patients), (C) 25 μM ibrutinib (19 patients) or (D) 50 μM idelalisib (16 patients). **(E-F)** CLL cells were co-cultured with autologous neutrophils at different N:CLL ratios for 24 h, in the presence or absence of (E) 25 μM Ibrutinib (10 patients) or (F) 50 μM Idelalisib (10 patients). The percentage of viable CLL cells (Annexin V-/PI-) was measured by double staining with annexin V–FITC and PI, followed by flow cytometric analysis. Dashes represent the mean of all patients and each dot represents the mean of triplicate for each patient. One-way ANOVA statistical test was used for multiple comparisons applying Holm-Sidak method.*p≤ 0.05, **p≤ 0.01, ***p≤ 0.001.

To investigate the effect of neutrophils on primary leukemic cells, freshly isolated CLL cells were cultured alone or with autologous neutrophils for 24 hours, in the presence or absence of different anti-leukemic agents. The percentage of viable CLL cells (Annexin V negative/PI negative) was measured by double staining with Annexin V–FITC and PI, followed by flow cytometric analysis. As shown in Figure 6A, vincristine reduced the percentage of viable CLL cells, an effect which was significantly inhibited in the presence of autologous neutrophils. Similar results for neutrophil-induced protection were obtained in the presence of fludarabine (Figure 6B).

Since targeted cancer therapy is becoming a major modality of CLL treatment, we aimed to investigate whether neutrophils induced protection on primary leukemic cells in the presence of two recently approved targeted therapies, ibrutinib (a Bruton's tyrosine kinase inhibitor) and idelalisib (a phosphoinositide 3-kinase delta inhibitor). Results in Figure 6C and 6D show that the decrease of cell viability induced by ibrutinib and idelalisib was significantly inhibited by the presence of neutrophils. This neutrophil-mediated protection of primary leukemic cells against ibrutinib and idelalisib was dependent on the N:CLL ratio (Figure 6E and 6F).

### Correlation between neutrophil blood counts at diagnosis and outcome in follicular lymphoma patients

A series of 367 patients with previously untreated follicular lymphoma treated at the Lyon Sud Hospital were evaluated according to neutrophil counts at diagnosis. The neutrophil counts cut-off value chosen was the upper quartile, which distinguished patients with counts above the normal limit. The median of neutrophil values are 3.42 in the lower three quartiles (0.1-5.49, 274 patients) and 9.86 in the upper quartile (5.5-72.8, 93 patients).

**Figure 7:**
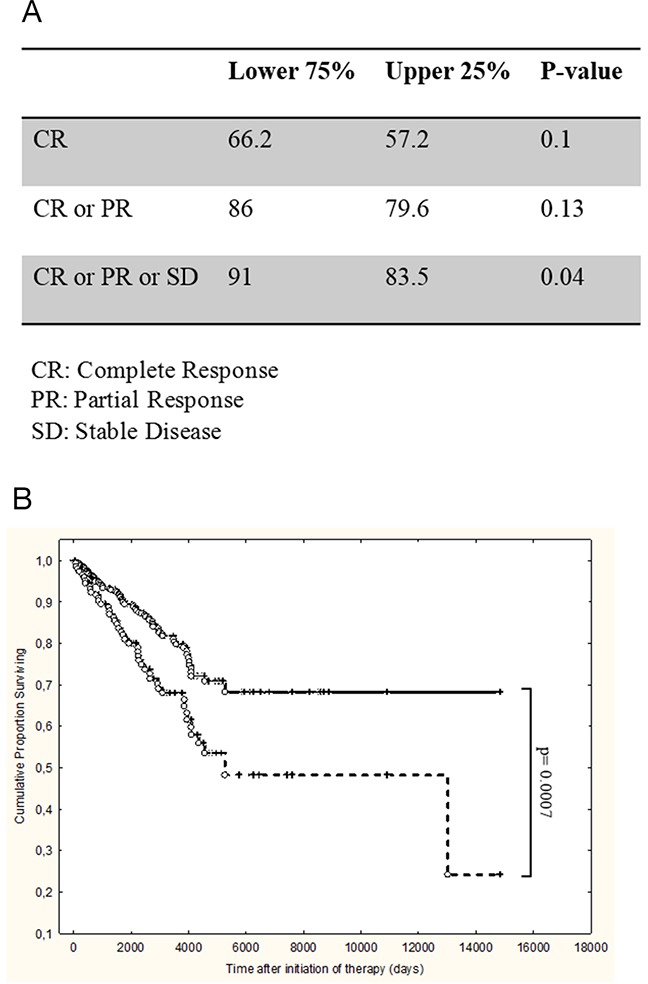
High levels of peripheral blood neutrophils at diagnosis are an adverse prognostic parameter in patients with follicular NHL 367 consecutive patients with follicular lymphoma were treated with R-CHOP regimen and analyzed for response and survival according to peripheral neutrophil values at diagnosis (upper quartile vs. lower quartiles). **(A)** Response according to neutrophil counts at diagnosis. CR: complete response; PR: partial response; SD: stable disease. **(B)** Overall survival according to neutrophil counts at diagnosis. Continuous line: lower quartiles, dotted line: upper quartile. p= 0,0007.

As shown in Figure 7, patients with high neutrophil counts at diagnosis had a significantly greater risk of not responding to therapy, with a trend towards less complete and/or partial responses (Figure 7). Additionally these patients had a significantly shorter overall survival (Figure 7, p=0.0007).

## DISCUSSION

Innate immune cells have been associated with tumor progression in patients and animal models of cancer [40]. Macrophages are important innate immune cells that have been shown to directly regulate tumor cell response to chemotherapy [23, 25]. However, the role of neutrophils, key players in the innate immune system, in regulating tumor response to anticancer treatment has been less studied. In the current study, we have shown that circulating neutrophils significantly reduce the sensitivity of malignant B-cells to a variety of anticancer therapies, thereby constituting yet a new mechanism through which the innate immune system could contribute adversely to the outcome in patients with lymphoma. While this may seem counter-intuitive given that chemotherapy induces neutrophil depletion, we emphasize that this effect is observed when tumor cells are exposed in the presence of neutrophils, while treatment-induced neutropenia is delayed several days after administration of treatment.

Tumor cells have previously been shown to influence the phenotype and properties of cells present in their microenvironment, in particular through the polarization of tumor infiltrating neutrophils [10, 41]. In our study, exposure to cytotoxic chemotherapy did not modify the expression levels of activation markers of neutrophils such as CD11a, CD11b, CD18, CD32 and CD66b, while their expression levels were significantly increased when neutrophils were co-cultured with RL cells. These results strongly suggest that NHL cells influence the phenotype and possibly the function of neutrophils with which they come into contact.

Neutrophil-induced protection on NHL cell proliferation in presence of chemotherapy was reduced in the presence of antibodies directed against ICAM-1 (expressed by NHL cells) or CD11b (expressed by neutrophils). The effect of blocking antibodies was only observed when each cell type was previously exposed to the specific antibody but not when the antibody was added to the co-culture, suggesting that the activation of ICAM-1 down-stream signaling pathways occurred rapidly, as has previously been described [30]. ICAM-1 is an adhesion molecule which has been shown to bind to several ligands including LFA-1 (CD11a/CD18), Mac-1 (CD11b/CD18) and CD11c/CD18. While it was originally suggested that ICAM-1 essentially played an adhesive role, evidence indicates that it is involved in the regulatory process of cell proliferation [30, 31]. Signal transduction by cell surface receptors is regulated by changes in the activity of specific kinases such as Raf/MAPK, which have been shown to mediate signaling functions including proliferation and cell survival [42, 43]. Thus we hypothesized that the interaction between ICAM-1 and CD11b in our system activates intracellular pathways in malignant B-cells allowing their enhanced proliferation in the presence of both neutrophils and anticancer agents. Additionally, co-culture of RL cells in the presence of neutrophils and vincristine up-regulated the expression of CD44 by the lymphoma cells. Blocking CD44 was also found to reverse neutrophil-induced protection of RL cell proliferation against vincristine. CD44 is another adhesion molecule involved in cell proliferation [32, 33].

The effect of neutrophils and/or vincristine on the expression of selected anti-apoptotic proteins by lymphoma cells differed significantly when cells were exposed to neutrophils or vincristine separately or simultaneously. While vincristine increased the content of Bcl-2, neutrophils alone did not induce a significant modification. Conversely, neutrophils together with vincristine strongly increased the expression of Mcl-1 in RL cells. Using the specific inhibitor of Mcl-1 MIM-1, we found that neutrophil-induced protection against vincristine was Mcl-1-dependent as it could be strongly reduced in the presence of MIM-1. Puthier *et al.* [35] have described apathway leading to Mcl-1 upregulation in human myeloma cells by IL6, a cytokine which is known to be released by activated neutrophils [44], probably through activation of the Jak/Stat pathway. Our results showed a detectable release of IL6 by neutrophils only when RL cells were co-incubated with vincristine and neutrophils simultaneously. Furthermore, blocking the IL6 receptor or JAK2 activation significantly decreased p-Stat-3 and Mcl-1 upregulation. Supplementary Figure 10 represents a schematic model which summarizes some possible mechanisms for neutrophil-induced protection of lymphoma cells against chemotherapy.

In order to evaluate the relevance of the protective effect of neutrophils in more complex models, we developed a microsphere co-culture system using differentiated promyelocytic cells. We also performed xenograft experiments with co-injection of RL cells and human neutrophils in immune-compromised mice. Both systems allowed us to confirm the protective effect against vincristine-induced cytotoxicity in the RL NHL model.

The clinical relevance of our observations was supported by two lines of results. First, using samples collected from CLL patients, we obtained a similar protective effect by autologous peripheral blood neutrophils on primary CLL cells, by reducing their sensitivity to several anti-leukemic agents. CLL cells are a subtype of lymphoproliferative disease of a particular interest since it allowed us to test recent targeted therapies which aim the BCR signaling pathway. Second, a study performed on a retrospective consecutive series of 367 patients with previously untreated follicular NHL showed that patients with higher than normal neutrophil blood counts at diagnosis had a lower probability to respond to treatment and had a significantly worse overall survival. This latter result is in keeping with the recent observation by Miao et al. who reported that patients with high neutrophil to lymphocyte ratios in peripheral blood displayed reduced response and survival after having received platinum-based therapy for ovarian cancer [45]. An issue relative to peripheral blood neutrophils, as compared to myeloid derived suppressor cells, is their relevance in terms of actual physical contact with tumor cells. The neutrophil infiltrate is highly variable according to lymphoma subtypes, with very dense infiltrates in some forms of Hodgkin's disease and reduced infiltrates in several forms of NHL. Conversely there is a consistent presence of mature neutrophils in the bone marrow, which is a frequent site of lymphoma localization with a relative resistance to therapy. In the case of myeloma, Ramachandran et al. recently showed that bone marrow neutrophils were involved in protection of multiple myeloma cells from chemotherapy [46].

In conclusion, using *in vitro* (2D and 3D models) as well as *in vivo* and *ex vivo* approaches, we showed that cell-cell contact between peripheral blood neutrophils and malignant B cells protect the latter from the cytotoxic effect of anticancer agents used in the treatment of lymphoproliferative diseases. CD11b/ICAM-1 interaction is implicated in neutrophil-mediated protection of malignant B cells, in addition to CD44. This protective effect is dependent on the Mcl-1 anti-apoptotic protein. Taken together, our data present an intriguing insight into the relevance of neutrophils in cancer cell sensitivity to anticancer agents and suggest that pharmacological modulation, for example with anti-Mcl-1 compounds, could be used to enhance the effect of cytotoxic agents in patients with NHL. Additional studies, including analyses of tumor infiltrating neutrophils, are required to determine whether neutrophils effectively protect tumor cells from anticancer agents in patients.

## MATERIALS AND METHODS

### Cell preparation and flow cytometry

*Cell lines-* Human non-Hodgkin's lymphoma (NHL) cell lines (follicular lymphoma RL cells, Burkitt's lymphoma Raji cells and mantle cell lymphoma Granta and Es-moult cells) and acute promyelocytic leukemia HL60 cells were obtained from ATCC. All cell lines were grown in complete RPMI medium (RPMI 1640 supplemented with 10% fetal bovine serum (FBS), 2 mM-glutamine, 100 U/mL penicillin and 100 mg/mL streptomycin) and cultured at 37°C in humidified 5% CO_2_. The cells were tested for mycoplasma twice per month.

*CFSE cell labeling*- Lymphoma B cells at 10^7^ cells/mL were incubated in PBS- containing 0.1% BSA with 5 μM CFSE for 10 min at 37°C. Labeling was stopped by adding 1 volume ice-cold FBS with 5 minutes of incubation on ice. Then cells were washed three times and incubated at 37°C with complete RPMI medium. Experiments started 24 hours after CFSE labeling. The entire procedure was carried out in dark.

*Isolation of human peripheral blood neutrophils-* Human neutrophils were isolated from fresh blood of healthy donors supplied by Etablissement Français du Sang (EFS) (Lyon, France) or from patients diagnosed with chronic lymphocytic leukemia (CLL). Ficol was carefully added to the diluted blood without mixing the phases. After a short centrifugation step (300 g for 35 minutes) at room temperature (RT), neutrophils together with red blood cells (RBCs) sediment at the bottom of the centrifuge vial. Neutrophils were extracted by 1:1 addition of 3% dextran in 0.9% NaCl while RBCs were removed by red cell lysis buffer. Neutrophils then washed and checked for their purity using a mixture of fluorochrome-conjugated purified monoclonal antibodies (Supplementary Antibodies Table 3 in supplementary data Neutrophils purity range was 90-98 % in all experiments.

*Flow cytometric analysis of surface antigens*- Cells were collected and washed in PBS with 4% FBS. Cells were incubated with fluorochrome-conjugated purified monoclonal antibodies at 4°C for 30 minutes. Later, cells were washed with PBS-4% FBS and the relative levels of surface antigens were assessed by FACS analysis on LSRII (BD Biosciences (San Jose, CA, USA)) using FACS Diva or Flowjo softwares.

### Antibodies and reagents

The detailed lists of reagents and antibodies used in our study are provided in the Supplementary data (Reagents and Supplementary Antibodies Tables 1, 2 and 3).

### Cell division and cell death assays

Cell division and death were evaluated using flow cytometry. Briefly, CFSE-labeled lymphoma B-cells (2×10^5^ cells/mL) were cultured alone or with fresh human neutrophils isolated from healthy donors at different neutrophil to tumor cell (N:T) ratios. Chemotherapeutic molecules (vincristine, doxorubicin, bortezomib, cisplatin, mafosfamide) were added to the culture system at different concentrations. After 48 hours, cells were collected and lymphoma cells were labeled with anti-CD19 antibody then analyzed for cell proliferation and cell death using CFSE and DAPI assays, respectively, on LSRII flow cytometer (Diva software).

In some experiments, 1 μg/mL anti-CD44, 10 nM ABT-199, 10 μM MIM-1, 1 μM AZD1480 (Jak2 inhibitor), 2 μg/mL tocilizumab (humanized monoclonal antibody against the IL6 receptor), 10 nM GD0973 (MEK inhibitor), 100 ng/mL TACI-Fc (blockade of BAFF/APRIL activity) were added to the culture system. In other experiments, CFSE-labeled RL cells and neutrophils were pre-incubated with blocking antibodies for 1 hour before co-culture. RL cells were incubated with 12 μg/mL anti-ICAM-1/CD54, 10 μg/mL anti-CD11a or anti-CD11c whereas neutrophils were incubated with 10 μg/mL anti-CD11a, anti-CD11b or anti-CD11c. Both cell types were then co-cultured for 48 hours in the presence of 10 nM vincristine.

### Cell uptake of doxorubicin

RL cells were incubated alone or with neutrophils at a ratio of N:T 10:1, in the presence or absence of 1 μM doxorubicin. After 2 hours of incubation, cells were collected on ice and washed with cold PBS then labeled with anti-human CD19 (for 1 hour on ice). Mean doxorubicin-related fluorescence in RL cells was collected by flow cytometry.

### Transwell and conditioned medium experiments

In transwell experiments, CFSE-labeled RL cells and neutrophils were cultured on opposite sides of microporous polyethylene terephthalate inserts (pore size of 0.4 μm). 2×10^6^ neutrophils were placed inside the inserts whereas 2×10^5^ CFSE-labeled RL cells were placed in the wells, in the presence or absence of 10 nM vincristine. After 48 hours, the proliferation and cell death fraction of RL cells were determined.

In conditioned medium (CM) experiments, neutrophils were incubated alone or with RL cells at a N:T ratio of 10:1, in the presence or absence of 10 nM vincristine for 2 hours. Then cells were harvested, washed and incubated with fresh complete RPMI medium. After another 2 hours of incubation, the supernatants (CM) were collected. Naïve CFSE-labeled RL cells were incubated with 100% of CM and compared to RPMI medium as a control, in the presence of 10 nM vincristine for 48 hours and proliferation and cell death of RL cells were determined.

### Expression microarray studies

RL cells were cultured alone or together with neutrophils at a N:T ratio of 10:1, in the presence or absence of 10 nM vincristine for 2 hours at 37°C. RL cells were negatively purified (≥ 90 % pure) after labeling the cells with anti-human CD15-Biotin (Miltenyi Biotec) then anti-Biotin Microbeads (Miltenyi Biotec) followed by magnetic column separation (LD Columns, Miltenyi Biotec). RNA was extracted using Qiamp RNeasy mini kit (Qiagen). After RNA amplification with total prep RNA amplification kit (Ambion), cRNA was hybridized on human HT-12 v4 beadchip (Illumina). Chips were scanned with an Illumina iScan and data was analyzed using GeneSpring and Ingenuity softwares. Microarray data have been deposited in the GEO database under accession number GSE69255.

### Intracellular protein content

RL cells were cultured alone or with neutrophils at a N:T ratio of 10:1, in the presence or absence of 10 nM vincristine. The intracellular labeling of anti-apoptotic Bcl-2 family members (Bcl-xL, Bcl-2 and Mcl-1) was performed by flow cytometry after 48 hours of incubation and at different times for phospho-Stat3, after initial labeling with anti-CD19, then fixation/permeabilization (BD Cytofix/Cytoperm kit) and staining with the appropriate antibodies.

### IL6 measurement in culture supernatants

IL6 was measured in our culture supernatants by means of ELISA according to manufacturer's protocols, with results expressed as pg/ml.

### Induction of human promyelocytic (HL60) cells granulocytic differentiation

HL60 cells were maintained at a logarithmic growth rate prior to induction of differentiation. HL60 cells (3×105 cells/mL) were induced to differentiate along the granulocyte pathway by 1 μM retinoic acid (RA, ref R2625, Sigma-Aldrich) and 1.25 % DMSO for 5 days. Medium was renewed on day 2 in the presence of RA and DMSO at the same concentrations.

Differentiation of HL60 cells was analyzed by two different parameters: cell-surface markers expression and morphological changes. On day 5, the cells were collected and HL60 or differentiated HL60 (HL60diff) cells were incubated with anti-human CD11b and anti-human CD38 for cell-surface markers expression analysis. In addition, cells were stained for morphological analysis using Kit RAL 555 Modified Giemsa staining kit (ref CB361550-0000, Cosmos Biomedical).

### 3-dimensional (3D) culture

For 3D experiments, 50,000 RL cells were incubated in Matrigel according to the manufacturer's protocol, in the presence or absence of differentiated HL60 cells (HL60*diff*) at a ratio of 10 HL60*diff*: 1 RL. 3D cultures were maintained for 7 days and culture medium was changed every two days. On day 5, vincristine was added at a concentration of 10 nM. On day 7, pictures were taken using DMI3000 fluorescent microscope at a 40x magnification then spheroids were dissociated by agitation in ice-cold PBS-5mM EDTA for 1 hour. Cells were then labeled with anti-human CD19 and anti-human CD38 and stained with Annexin V–FITC/PI using Annexin V-FLOUS staining kit followed by flow cytometry, to determine the proportion of viable (CD19 positive AnnexinV negative/PI negative) RL cells.

### Animal studies

All animal procedures were performed in accordance with the European Union Directive (86/609/EEC). Experiments were performed under individual permit and in animal care facilities accredited by the French Ministry of Agriculture. The study was approved by the local animal ethical committee.

Studies were conducted using female SCID/CB17 mice (4 weeks old, 5 mice/group, Charles River). Mice were injected subcutaneously with 3×10^6^ RL cells alone or with freshly human neutrophils isolated form peripheral blood of healthy donors at a N:T ratio of 5:1. Vincristine was injected intraperitoneally at a dose of 1 mg/kg on the same day. Tumor growth was monitored by caliper measurement of the tumor volume every 3-4 days.

### Analysis of patient samples and clinical data

To determine the clinical relevance of our findings in patients with lymphoid malignancies, we analyzed two models: the protective effect of autologous neutrophils on CLL cells ex vivo, and the correlation between peripheral neutrophils counts and patient outcome in patients with follicular lymphoma.

Peripheral blood on EDTA was obtained from patients (aged 58- 90 years) diagnosed with CLL according to standard criteria. Procedures were conducted under the approval of Lyon Hospital Ethics committee with all patients signing informed consent. CLL cells were isolated by Ficoll density-gradient centrifugation and purity was verified by CD19 labeling (≥ 90% CD19-positive). CLL cells (2×10^5^ cells/mL) were cultured alone or with autologous neutrophils, isolated as described above, at different N:CLL ratios. Chemotherapeutic agents (vincristine and fludarabine) or targeted therapies (ibrutinib and idelalisib) were added to the cultures. After 24 hours, the percentage of viable CLL cells (Annexin V negative/PI negative) was determined by double staining of the cells with Annexin V–FITC/PI.

Correlations between neutrophil blood counts at diagnosis and clinical outcome in patients with follicular lymphoma were determined on a retrospective series of 367 consecutive patients receiving their first line treatment in the Hematology Department of the Hospices Civils de Lyon. Initial and follow-up data were collected from routine hospital charts according to French Ethics Requirements.

### Statistics

Flow cytometry data were analyzed using BD FACSDiva™ software and FlowJo, LLC data analysis software. The sample size (n) for each experimental group/condition represent biological replicates and indicated in each figure legend. Results were expressed as the mean ± SD of at least three independent experiments, each performed in triplicates. One Way Anova test (Sigma Plot^®^, Systat Software) and Student's *t* test were used for comparison among groups, and p- values were reported. Some experiments were performed once in triplicate. For *in vivo* experiments, results were expressed as median ± S.E.M. Response to treatment according to international response criteria [47] were compared using the Chi2 test. Comparison of overall survival data of patients were assessed using the Log-Rank test.

## SUPPLEMENTARY MATERIALS FIGURES AND TABLES


