# Supplementary material for: Neutrophils protect lymphoma cells against cytotoxic and targeted therapies through CD11b/ICAM-1 binding
Source: Oncotarget. 2017 Aug 18;8(42):72818–34. doi: 10.18632/oncotarget.20350 (PMC5641171; doi:10.18632/oncotarget.20350)
Supplement: Supplementary file 1 [file oncotarget-08-72818-s001.pdf]

Neutrophils protect lymphoma cells against cytotoxic and targeted therapies through CD11b/ICAM-1 binding

SUPPLEMENTARY MATERIALS

Reagents

Chemotherapeutic agents: Vincristine was obtained from EG labo (Boulogne-Billancourt, France), doxorubicin from Accord (Paris, France), bortezomib from Janssen (Issy-les-Moulineaux, France), cisplatin from Mylan (Saint Priest, France), mafosfamide from Santa Cruz (Dallas, TX, USA) and fludarabine from Schering (Kenilworth, NJ, USA).

Targeted therapies: Bruton's tyrosine kinase (Btk) inhibitor (ibrutinib) from CliniSciences (Nanterre, France) and phosphoinositide 3-kinase (PI3Kdelta) inhibitor (idelalisib) from Santa Cruz (Dallas, TX, US).

Inhibitors: ABT-199 was obtained from ChemieTek (Indianapolis, IN, USA) MIM-1 from Tocris bioscience (Missouri, USA), AZD1480 from InvivoGen (San Diego, CA, USA) and GD-0973 from Genentech (San Francisco, CA, USA).

Others: Human IL-6 ELISA kit was from invitrogen (Carlsbad, CA, USA). Red cell lysis buffer (ref 555899), BD Matrigel basement membrane matrix (ref 354234) and BD Cytofix/Cytoperm Fixation/Permeabilisation kit (ref 554715) were from BD Biosciences (San Jose, CA, USA). Ficoll (Pancoll, ref P04-60500) obtained from PAN Biotech (Aidenbach, Germany). Dimethyl sulfoxide (DMSO, ref D8418), dextran (ref D8906), ethylenediaminetetraacetic acid (EDTA, ref E5134) and bovine serum albumin (BSA, ref A7906) were from Sigma-Aldrich (Saint-Quentin-Fallavier, France). 4',6-diamidino-2-phenylindole (DAPI, ref 6484) and Annexin V-FLOUS staining kit (ref 11 988 549 001) were from Roche (Boulogne-Billancourt cedex, France). Sodium chloride (NaCl, ref S3014) was form Euromedex (Souffelweyersheim, France). RPMI 1640 (ref 21875-034), fetal bovine serum (FBS, ref 10270-106) and phosphate-buffered saline (PBS, ref 14040-091) were from Gibco Invitrogen (Carlsbad, CA, USA). CarboxyFluorescein diacetate succinimidyl Ester (CFSE, ref C34554), N-acetyl-L-alanyl-L-glutamine (L-Glutamine, ref 25030-024) and penicillin streptomycin (Pen Strep, ref 15140-122) were from Life technologies. Millicell cell culture PET inserts (24-well Millicell, ref PIHT12R48) were from Millipore (Île-de-France, France).

Antibodies

Supplementary Antibodies Table 1: Blockade of receptor-ligand interactions

Antigen	Clone	Antibody supplier
CD11a	HI111	Biolegend
CD11b	ICRF44	Biolegend
CD11c	3.9	Biolegend
CD44	A3D8	Sigma-Aldrich
ICAM-1/CD54	BBIG-I1	R and D systems
IgG1	X40	BD Biosciences
HCA257 (tocilizumab)	AbD22155_hIgG1	AbD Serotec
Human TACI/TNFRSF13B		R and D systems

Supplementary Antibodies Table 2: Fluorochrome-conjugated purified monoclonal antibodies

Antigen	Fluorochrome	Clone	Antibody supplier
CD11a	PE	G43-25B	BD Biosciences
CD11b	BV650	M1/70	Biolegend
CD15	Alexa Fluor 700	W6D3	Biolegend
CD15	FITC	VIMC6	Miltenyi biotech
CD18	BV421	6.7	BD Biosciences
CD19	APC	SJ25C1	BD Biosciences
CD19	PE-Cy7	SJ25C1	BD Biosciences
CD32	PE	3D3	BD Biosciences
CD38	APC	HB7	BD Biosciences
CD64	APC	10.1	BD Biosciences
CD66b	PerCP/Cy5.5	G10F5	Biolegend
BCL-2	PE	Bcl-2/100	BD Biosciences
Bcl-xL	Alexa Fluor 488	54H6	Cell signaling
Mcl-1*	APC	Poly6136	Biolegend
Phospho-Stat3 (Tyr705)	PE	D3A7	Cell signaling

*Purified anti-Mcl-1 antibody was labeled with APC fluorochrome using LYNX Rapid APC Antibody Conjugation kit (Bio-Rad).

Supplementary Antibodies Table 3: Fluorochrome-conjugated purified monoclonal antibodies for neutrophils purity. All antibodies were obtained from Miltenyi biotech

Antigen	Fluorochrome	Clone
CD3	VioBlue	BW264/56
CD14	APC-Vio770	TÜK4
CD15	APC	VIMC6
CD16	FITC	VEP13
CD19	PE-Vio770	LT19
CD45	PerCP-Vio700	5B1
CD56	PE	AF12-7H3

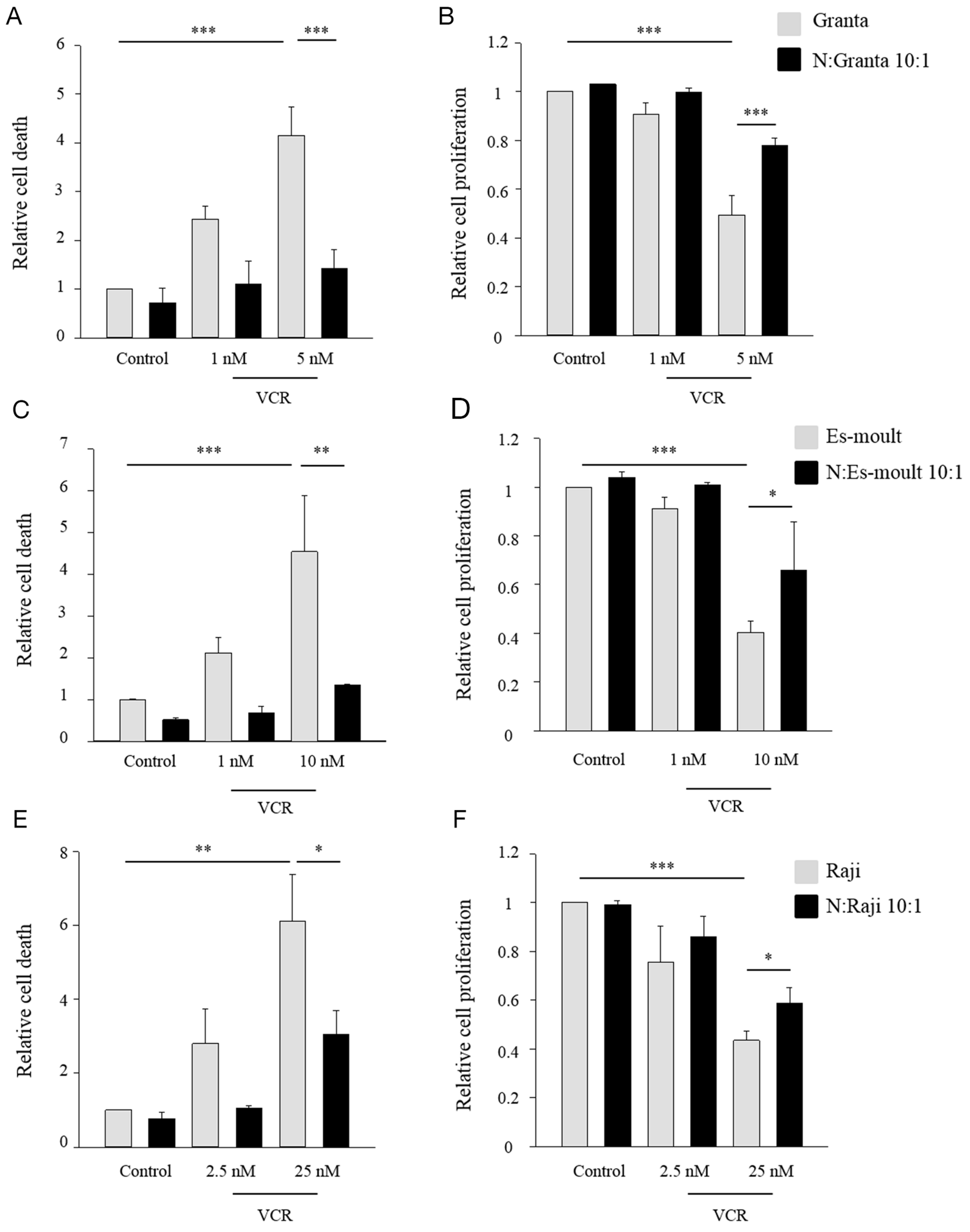

Supplementary Figure 1: Neutrophils protect lymphoma B cells against vincristine. CFSE-labeled lymphoma B cells were cultured alone or together with neutrophils (N) at N:T ratio 10:1, in the presence or absence of different concentrations of vincristine (VCR). After 48 h of incubation, cells were labeled with anti human-CD19 then resuspended in DAPI (2 μg/ml) followed by flow cytometric analysis. Cell death **(A, C, E)** and cell proliferation **(B, D, F)** of CD19 positive population were measured using DAPI and CFSE assays, respectively. Data are expressed as mean ± SD of at least two independent experiments performed in triplicates. The data are presented relative to the control. One-way ANOVA statistical test was used for multiple comparisons applying the Holm-Sidak method. *p≤ 0.05, **p≤ 0.01, ***p≤ 0.001

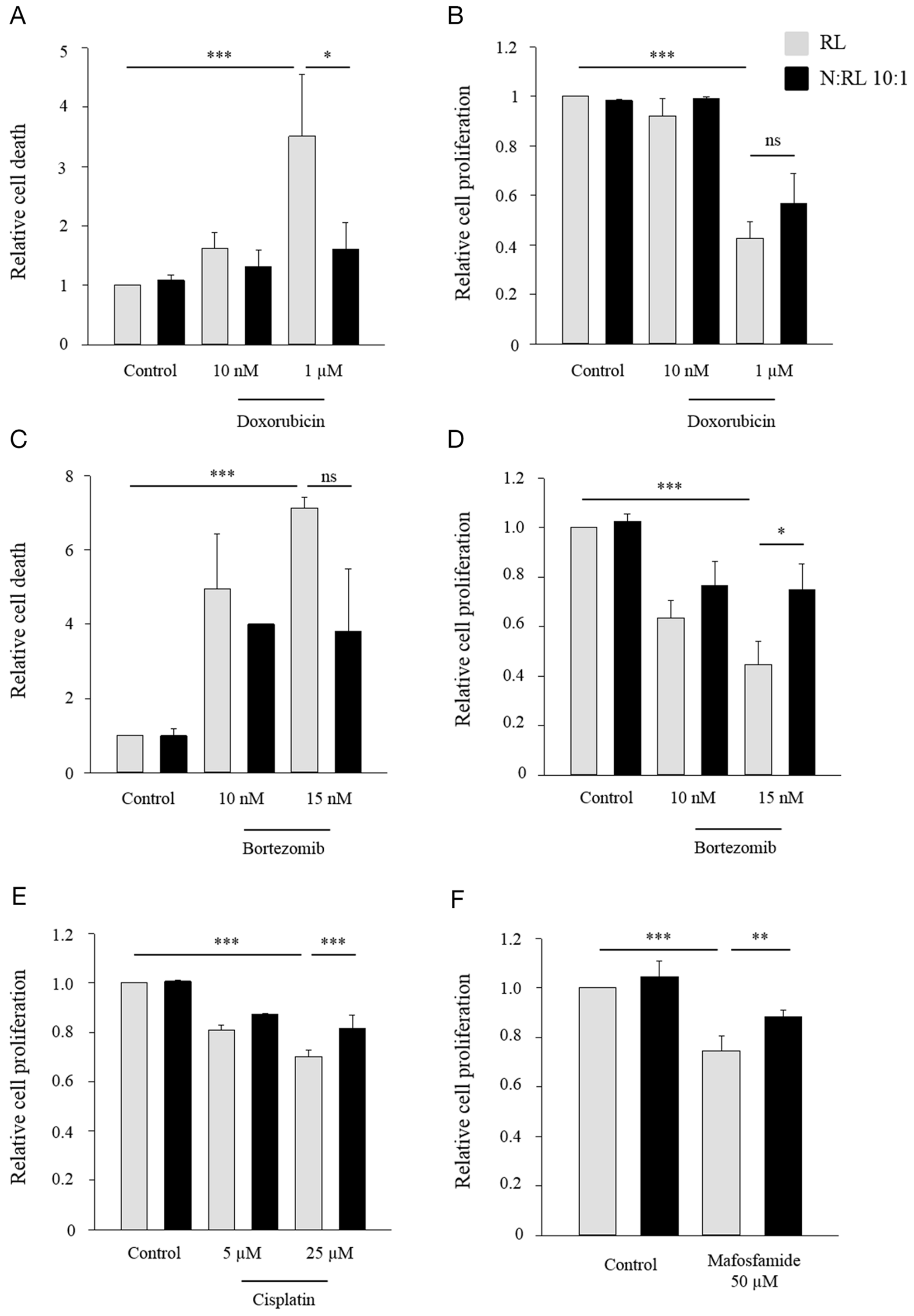

Supplementary Figure 2: Neutrophils protect RL lymphoma cells against chemotherapeutic agents. CFSE-labeled RL cells were cultured alone or together with freshly purified human neutrophils (N) at N:T ratio 10:1, in the presence or absence of different chemotherapeutic agents. After 48 h of incubation, cells were labeled with anti human-CD19 then resuspended in DAPI (2 µg/ml) followed by flow cytometric analysis. Cell death **(A, C)** and cell proliferation **(B, D, E, F)** of CD19 positive population were measured using DAPI and CFSE assays, respectively. Data are expressed as mean ± SD of at least three independent experiments performed in triplicates. The data are presented relative to the control. One-way ANOVA statistical test was used for multiple comparisons applying the Holm-Sidak method. $*p \leq 0.05$, $**p \leq 0.01$, $***p \leq 0.001$, ns= not significant.

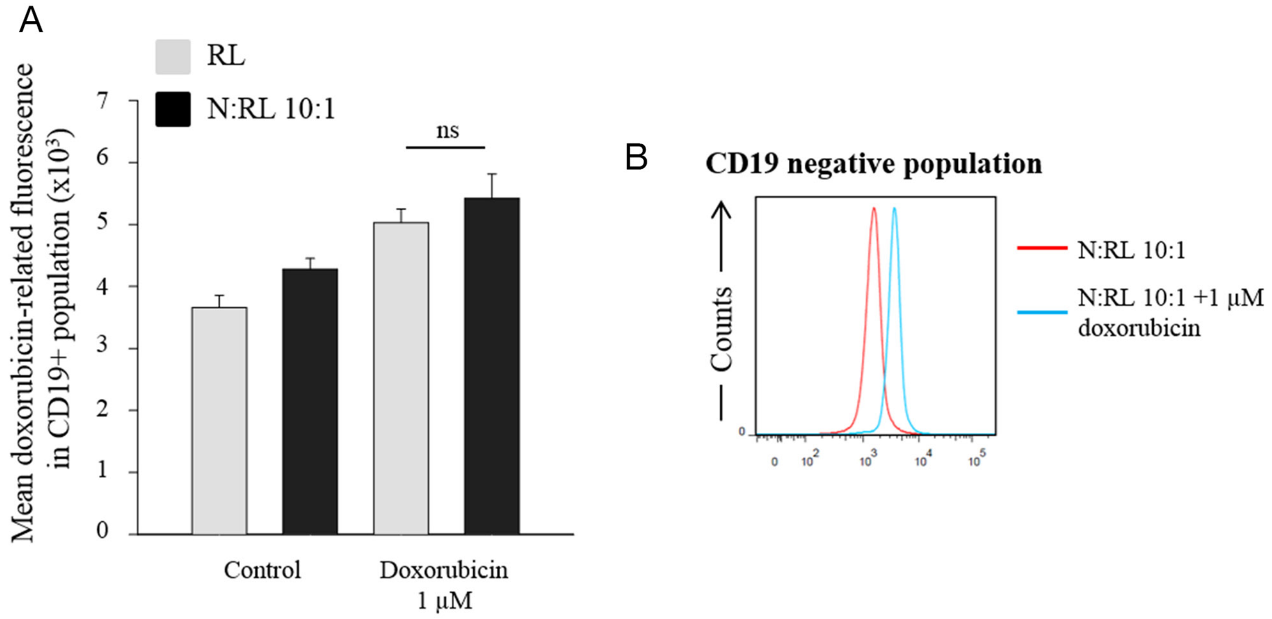

Supplementary Figure 3: Flow cytometry analysis of doxorubicin accumulation in RL cells and neutrophils. RL cells were cultured alone or together with neutrophils (N) at N:T ratio 10:1, in the presence or absence of 1 µM doxorubicin. After 2 hours of incubation, cells were labeled with anti human-CD19 followed by flow cytometric analysis. **(A)** CD19 positive population was analyzed for doxorubicin accumulation represented by FITC Mean Florescence Intensity (MFI). **(B)** Histogram reveals doxorubicin accumulation by CD19 negative population in the co-culture system. Data are expressed as mean ± SD of one experiment performed in triplicates. One-way ANOVA statistical test was used for multiple comparisons applying Holm-Sidak method. ns= not significant

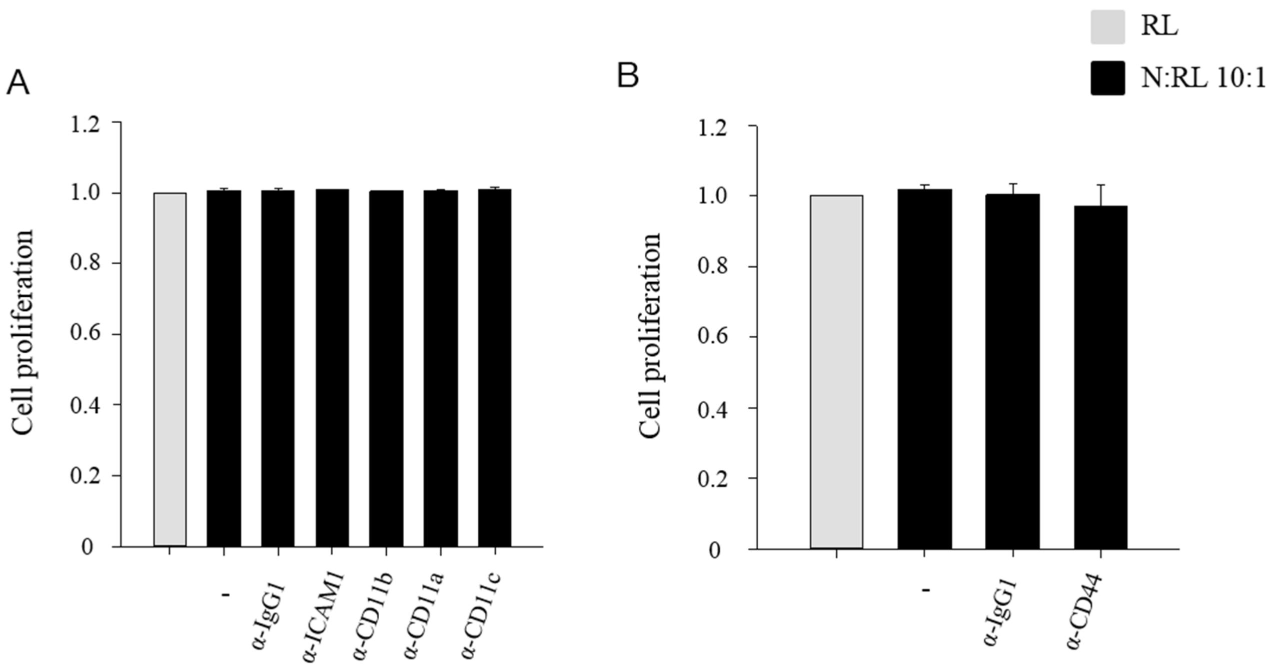

Supplementary Figure 4: Effect of blocking antibodies on RL cell growth in the absence of VCR. **(A)** CFSE-labeled RL cells were blocked against ICAM1 (12 µg/ml), CD11a or CD11c (10 µg/ml) and neutrophils were blocked against CD11a, CD11b or CD11c (10 µg/ml) for 1 hour. Anti-IgG1 served as a control. CFSE-labeled RL cells were cultured alone or together with neutrophils (N) at N:T ratio 10:1 for 48 h. Data are expressed as mean ± SD of three independent experiments. The data are presented relative to the control. **(B)** CFSE-labeled RL cells were cultured alone or together with neutrophils in the presence or absence of CD44 blocking antibody (1 µg/ml). Cell proliferation of CD19 positive population was measured using CFSE assay followed by flow cytometric analysis. Data are expressed as mean ± SD of three independent experiments. The data are presented relative to the control. One-way ANOVA statistical test was used for multiple comparisons applying the Holm-Sidak method.

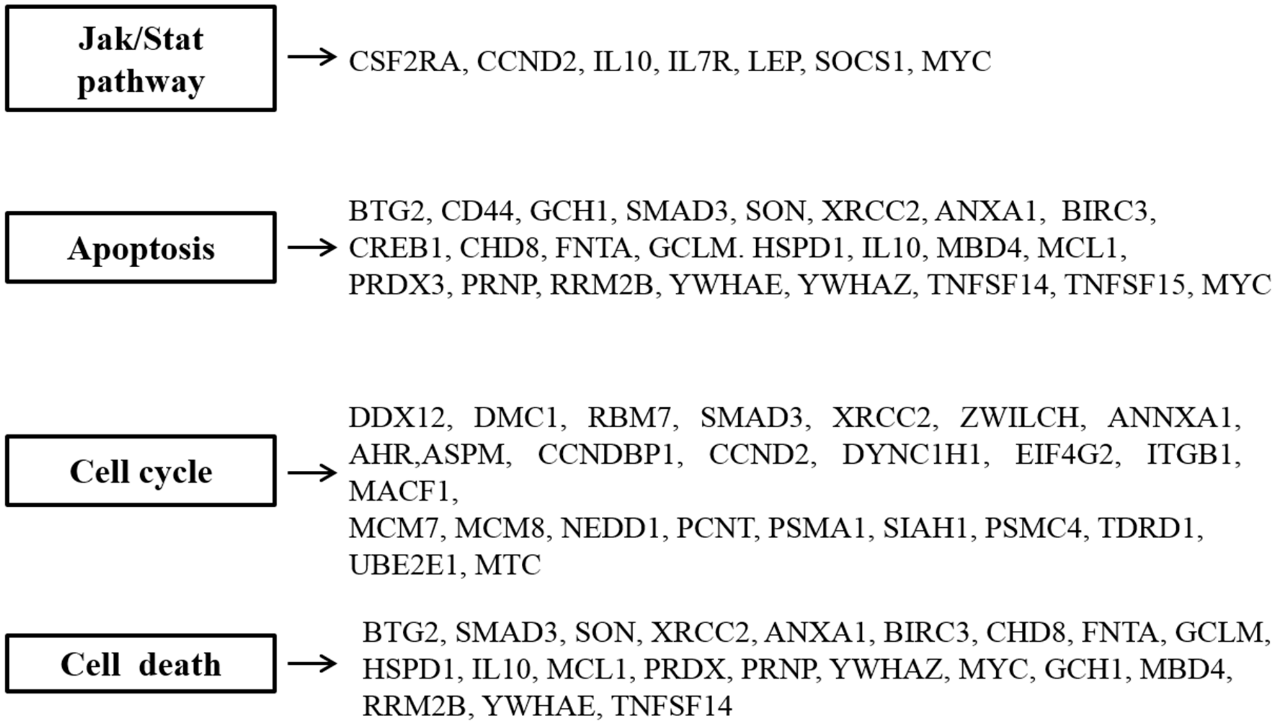

Supplementary Figure 5: Transcriptomic analysis of RL cells showed a regulation in multiple genes involved in several pathways. RL cells were cultured with neutrophils at a N:T ratio of 10:1, in the presence of 10 nM vincristine for 2 hours at 37^{0}C. RL cells were purified and RNA was extracted. cRNA was hybridized on human HT-12 v4 beadchip. Chips were scanned and data was analyzed.

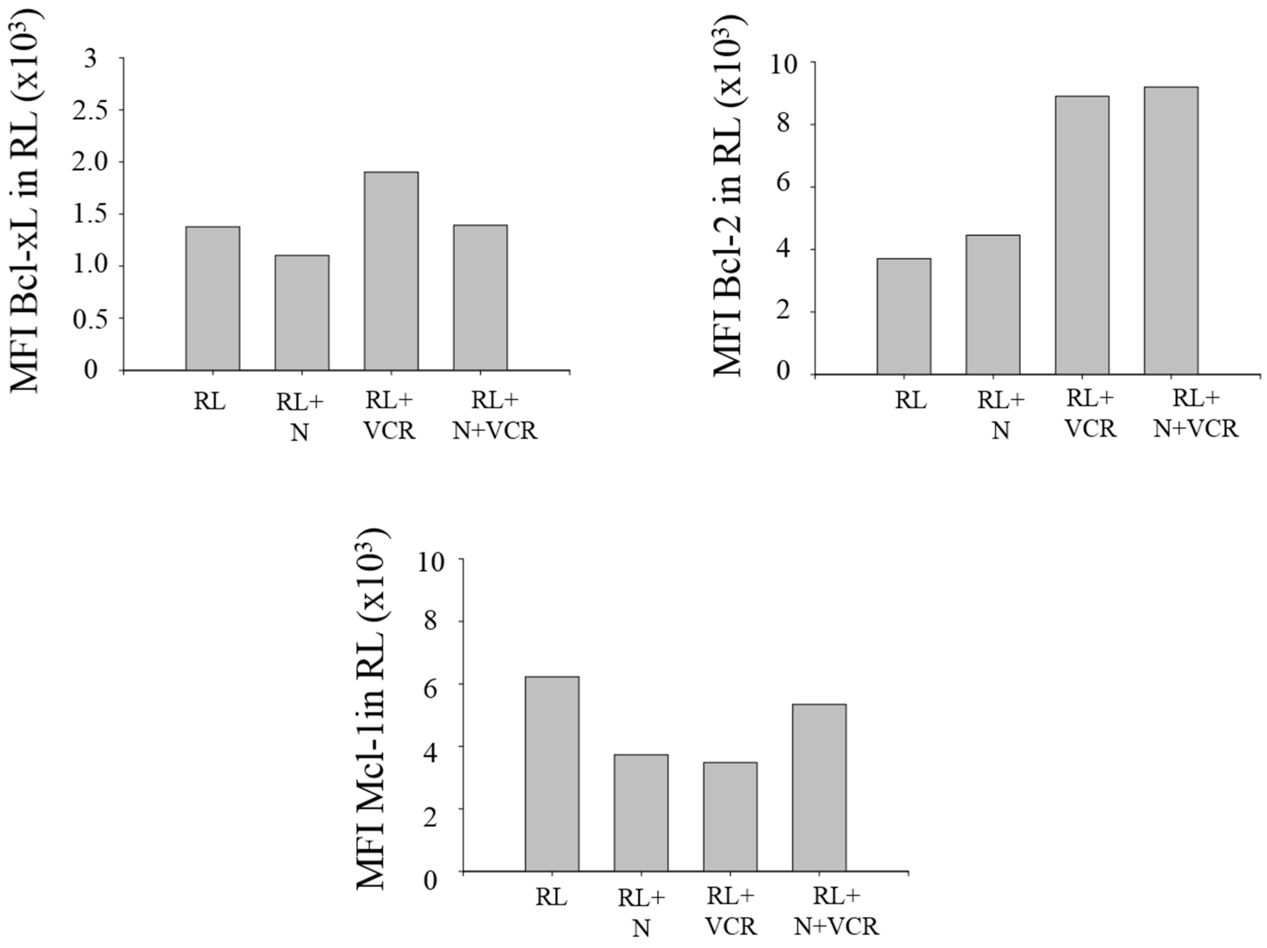

Supplementary Figure 6: Absolute expression of Bcl-2 family members (Bcl-xL, Bcl-2, Mcl-1) in RL cells. RL cells were cultured alone or together with neutrophils (N) at N:T ratio 10:1, in the presence or absence of 10 nM VCR for 48 h. Cells were collected and labeled with anti-human CD19 then fixed and permeabilized. Later, the intracellular labeling of anti-apoptotic Bcl-2 family members was performed by flow cytometry. Representative figure of three independent experiments.

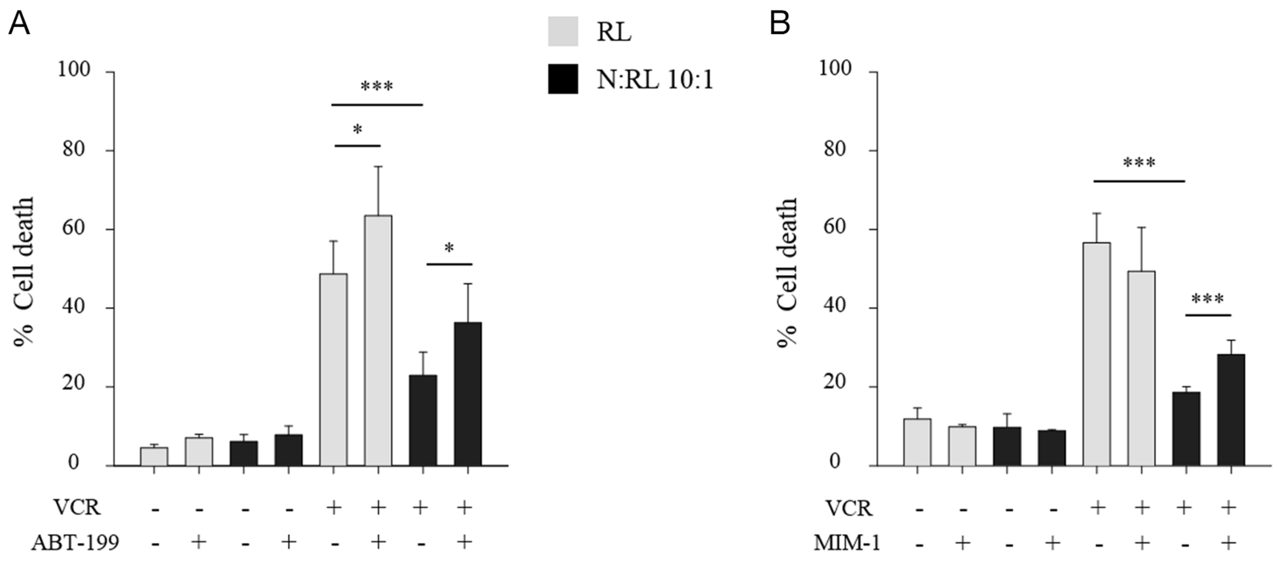

Supplementary Figure 7: Neutrophil-induced protection is dependent on Mcl-1 antiapoptotic protein. RL cells were cultured alone or together with neutrophils at N:T ratio 10:1, in the presence or absence of 10 nM VCR. ABT-199 (10 nM) **(A)** or MIM1 (10 µM) **(B)** inhibitors were added to the culture system. After 48h of incubation, cells were labeled with anti human-CD19 then resuspended in DAPI (2 µg/ml) followed by flow cytometric analysis. Cell death of CD19 positive population was measured using DAPI assay. Data are expressed as mean ± SD of three independent experiments performed in triplicates. One-way ANOVA statistical test was used for multiple comparisons applying the Holm-Sidak method. *p≤ 0.05, ***p≤ 0.001.

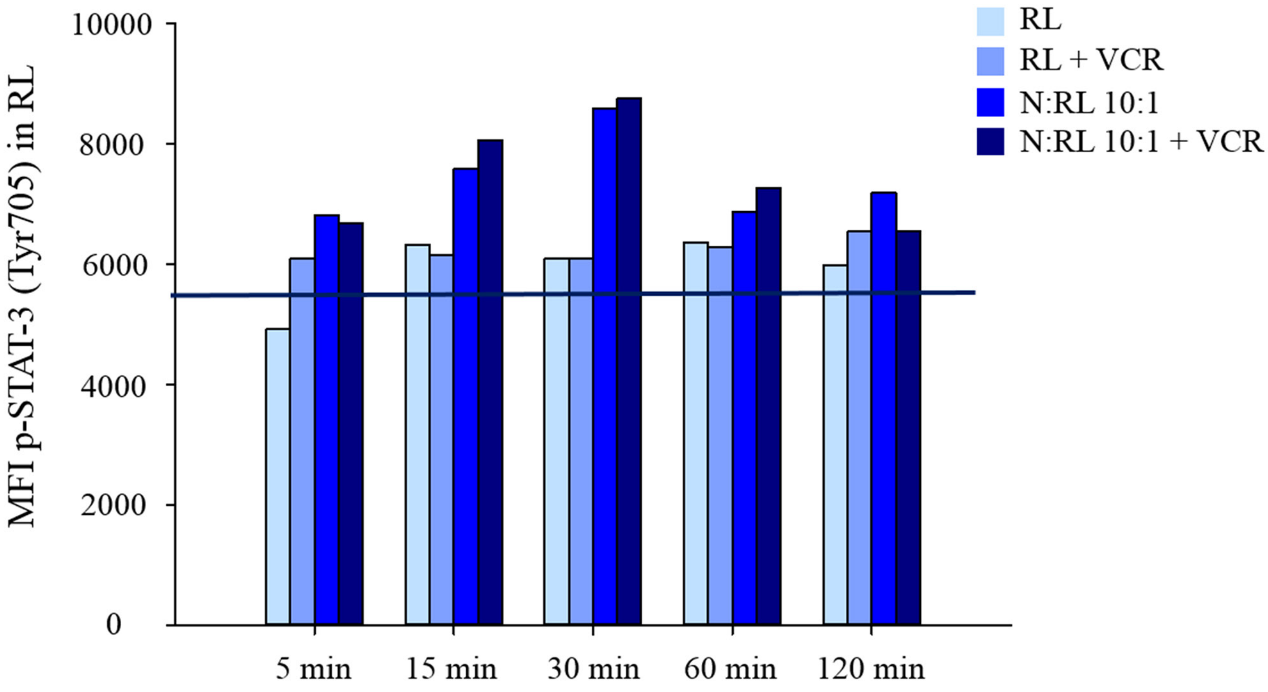

Supplementary Figure 8: Neutrophils induce constitutive Stat-3 phosphorylation in RL cells. RL cells were cultured alone or together with neutrophils (N) at N:T ratio 10:1, in the presence or absence of 10 nM VCR. At different times of incubation, the cells were collected and labeled with anti-human CD19 then fixed and permeabilized. Later, the intracellular labeling of p-Stat-3 (Tyr705) was performed by flow cytometry. Representative figure of two independent experiments.

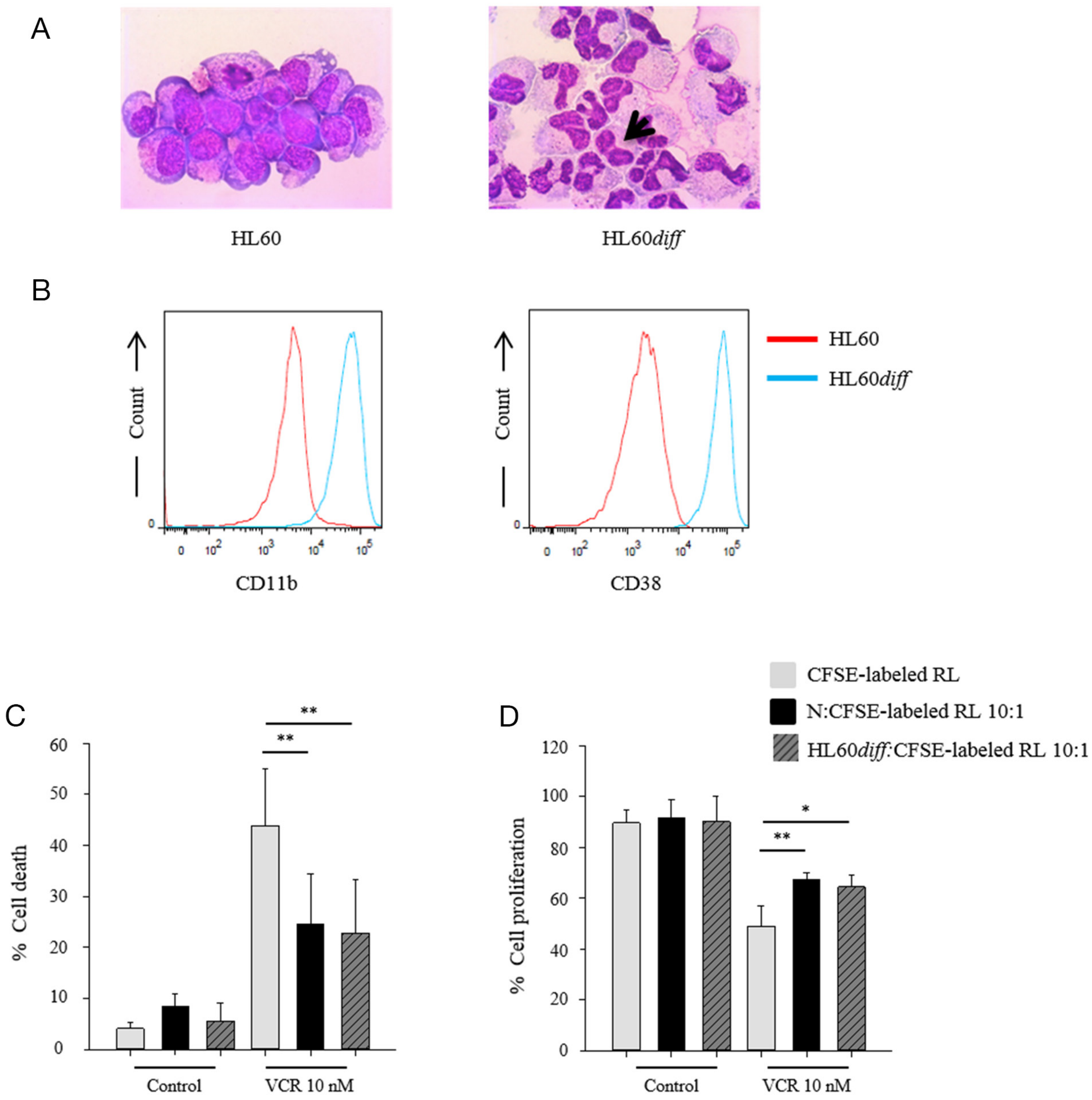

Supplementary Figure 9: Neutrophil-like HL60*diff* cells protect RL lymphoma cells against vincristine in 2D culture. **(A)** Morphological changes of HL60 cells.. Pictures were taken using Leica DMR-XA microscope with 100x magnification. Bold black arrow point the multi-lobed nucleus. Pictures are representative of five independent experiments. **(B)** HL60 and differentiated HL60 cells (HL60*diff*) cells were labeled with anti-human CD11b and anti-human CD38 antibodies followed by flow cytometry analysis. **(C-D)** CFSE-labeled RL cells were cultured alone or together with neutrophils (N) or HL60*diff* cells at N:T or HL60*diff*:T ratio 10: 1, in the presence or absence of 10 nM VCR. After 48 h of incubation, cells were labeled with anti human-CD19 then resuspended in DAPI (2 µg/ml) followed by flow cytometric analysis. Cell death (C) and cell proliferation (D) of CD19 positive population were measured using DAPI and CFSE assays, respectively. Data are expressed as mean ± SD of three independent experiments performed in triplicates. One-way ANOVA statistical test was used for multiple comparisons applying the Holm-Sidak method. $*p \leq 0.05$, $**p \leq 0.01$

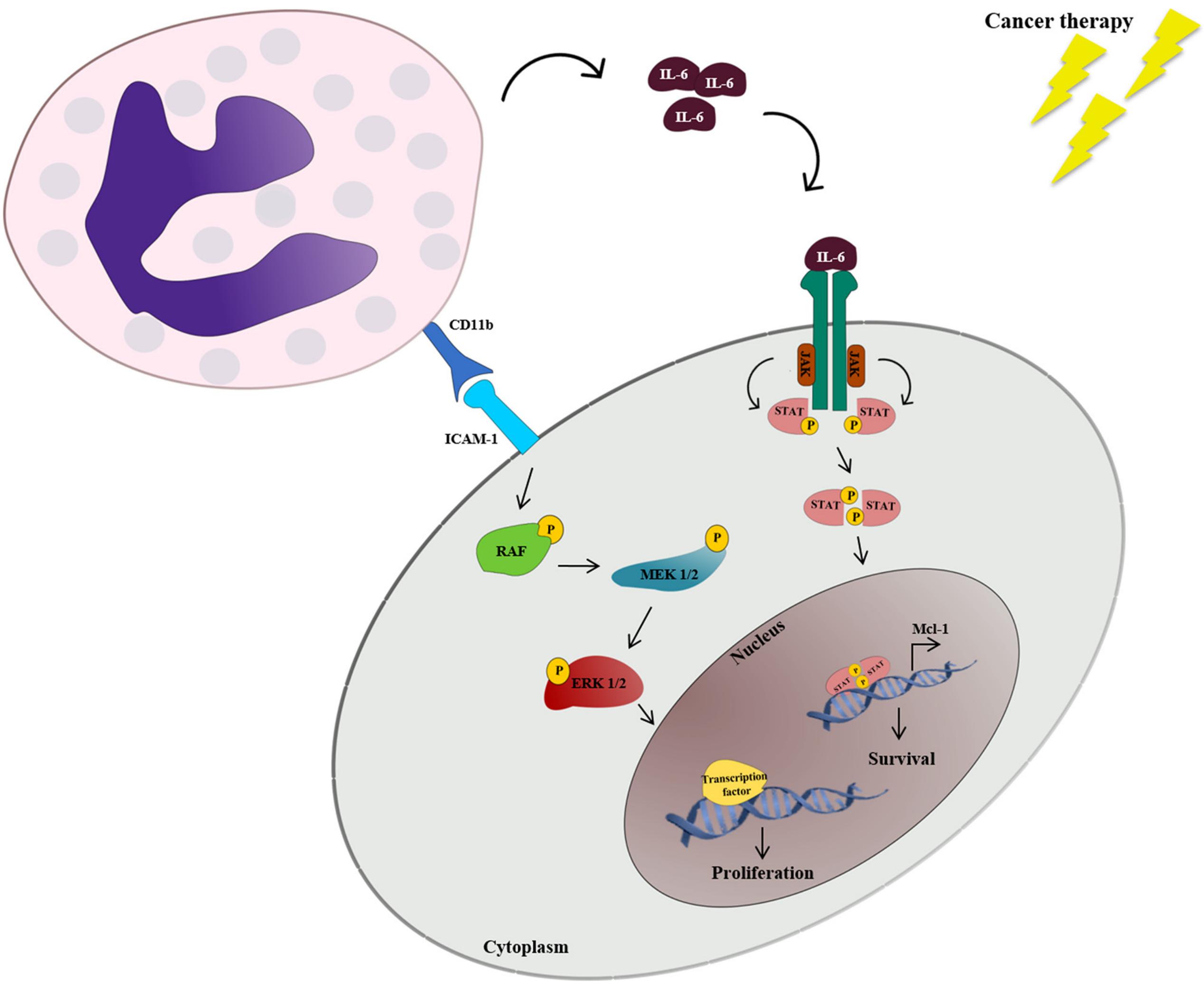

Supplementary Figure 10: Schematic model summarizes the possible mechanisms for neutrophil-induced protection on lymphoma cells against chemotherapy. Cell-cell contact between neutrophils and lymphoma cells through CD11b/ICAM-1 interaction leads to Raf-MEK-ERK pathway activation that results in enhancing cell proliferation. As well, direct contact between neutrophils and lymphoma cells induce IL6 secretion by the activated neutrophils. Then IL6 bind to their receptors results in Jak/Stat pathway activation and Mcl-1 upregulation enhancing lymphoma cellsurvival.

Supplementary Table 1: Mean fluorescence intensity of neutrophils activation markers. Neutrophils (N) were cultured alone or together with RL cells at N:T ratio 10:1 in the presence or absence of 10 nM VCR. After 48 h of incubation, cells were collected and labeled with anti-human CD15. Additionally, they were labeled with anti-human CD11a, CD11b, CD18, CD32, CD66b and CD64. Mean fluorescence intensity is depicted for CD15 positive population. Data are expressed as mean ± SD of three independent experiments. One-way ANOVA statistical test was used for multiple comparisons applying Holm-Sidak method. **p≤ 0.01, ***p≤ 0.001 (N vs. N:RL), #p≤ 0.05, ##p≤ 0.01, ###p≤ 0.001 (N vs. N:RL + 10 nM VCR).

Antigen	N	N + 10 nM VCR	N:RL	N:RL + 10 nM VCR
		mean ± SD		
CD11a	2380 ± 862	2122 ± 596.2	3266 ± 959	2955 ± 737
CD11b	4825 ± 1786	6407 ± 1280	35733 ± 3092***	37050 ± 5020###
CD18	10645 ± 3046	12250 ± 1202	45300 ± 707**	43600 ± 1697#
CD32	2168 ± 202	2404 ± 340.8	1420 ± 355	946 ± 241#
CD64	705 ± 94	731 ± 67	2380 ± 885	1893 ± 883
CD66b	4423 ± 1350	4764 ± 1075	23200 ± 7114**	18133 ± 6800##

Supplementary Table 2: Ratios of the most strongly down-regulated and up-regulated genes in RL lymphoma cells. RL cells were cultured with neutrophils at a N:T ratio of 10:1 in the presence of 10 nM vincristine for 2 hours at 37^0C. RL cells were purified and RNA was extracted. cRNA was hybridized on human HT-12 v4 beadchip. Chips were scanned and data was analyzed using GeneSpring and Ingenuity softwares

Downregulated genes			Upregulated genes		
	p-value	Ratio		p-value	Ratio
DAZ4	0,001	0,48	PPA2	0,001	1,75
LOC650867	0,001	0,52	ALPP	0,014	1,82
TCEA1	0,001	0,53	LRRC37B2	0,011	1,82
LOC644063	0,002	0,57	LOC100131718	0,006	1,89
BTG2	0,020	0,57	DYNC1H1	0,022	1,91
MEST	0,005	0,58	KLRB1	0,007	1,93
CCNC	0,008	0,60	LEP	0,021	1,93
SUMO2	0,014	0,75	SNORA45	0,001	1,97
DCK	0,015	0,75	TMEM137	0,041	2,06
MYC	0,029	0,77	CD44	0,007	2,06
